# Influence of the β-Casein Genotype of Cow’s Milk (A1, A2) on the Quality and β-Casomorphin-7 (BCM-7) Content of a Semi-Hard Cheese During Production

**DOI:** 10.3390/foods14030463

**Published:** 2025-02-01

**Authors:** Louisa Zinßius, Lucas Keuter, Carsten Krischek, Nadja Jessberger, Benedikt Cramer, Madeleine Plötz

**Affiliations:** 1 Institute of Food Quality and Food Safety, University of Veterinary Medicine Hannover, 30173 Hannover, Germany; 2 Institute of Food Chemistry, University of Münster, 48149 Münster, Germany; l_keut03@uni-muenster.de (L.K.); cramerb@uni-muenster.de (B.C.)

**Keywords:** bovine milk, semi-hard cheese, β-casomorphin-7 (BCM-7), A1-β-casein, A2-β-casein, physicochemical parameters, microbiological parameters

## Abstract

Cow’s milk contains A1- and A2-β-caseins. The breakdown of A1-β-casein produces β-casomorphin-7 (BCM-7), a peptide with opioid-like properties that is associated with health aspects. In addition, A1- and A2-β-casein have different technological properties. The aim of the present study was to investigate whether cheese produced from the milk of homozygous A1A1 and A2A2 cows varies in terms of its physicochemical parameters and BCM-7 concentration. These parameters were analyzed during initial cheese processing, six weeks of ripening and 84 days of storage, including additional microbiological analyses during the storage period. The pH values of the A1A1 cheeses were higher than those of the A2A2 cheeses from the beginning of production until the starter culture bacteria were added. The yellowness values of the A1A1 cheeses were lower until the salt bath treatment. Water activity, lightness, hardness, fat, protein, NaCl and dry matter content, as well as color and microbiological parameters, were not affected by the β-casein genotype. BCM-7 concentrations were higher in the A1A1 cheeses after pressing and during ripening. We found mainly comparable quality characteristics and slightly different BCM-7 levels in the A1A1 and A2A2 cheeses. From this point of view, both varieties are equally suitable for cheese production.

## 1. Introduction

Caseins constitute the majority of the protein fraction in bovine milk. Among these, αs1-casein is the most abundant, followed by β-casein, αs2-casein and κ-casein [1,2]. Other proteins present in cow’s milk include β-lactoglobulin, lactoferrin, serum albumin, α-lactalbumin and immunoglobulins [1]. The caseins show a high genetic polymorphism. Currently, 4 variants of αs2-casein (A, B, C, D), 8 variants of αs1-casein (A, B, C, D, E, F, G, H), 12 variants of κ-casein (A, B, B2, C, E, F1, F2, G1, G2, H, I, J) and 15 genetic variants of β-casein have been reported. The β-casein variants are named according to the order of discovery: A1, A2, A3, B, C, D, E, F, G, H1, H2, I, J, K, L, whereas A1- and A2-β-caseins are the most common genetic variants [1,2]. A1- and A2-β-casein proteins only differ by a single amino acid at position 67 of their primary structure: A1-β-casein contains histidine, while A2-β-casein contains proline at this position [2]. However, this amino acid substitution is associated with a varying secondary protein structure and, therefore, (functional) differences of the protein and the milk [1,2].

Most important is the formation of different degradation products, such as the beta-casomorphin (BCM) peptides. Among BCMs, β-casomorphin-7 (BCM-7), which consists of seven amino acids, has been extensively studied, as the consumption of milk containing this peptide has been associated with negative health effects [1]. Notably, enzymatic digestion of A1-β-casein and A2-β-casein results in different proteolytic patterns, with the first one containing significantly higher concentrations of BCM-7 [3]. This is attributed to the lower enzymatic resistance of the histidine–isoleucine bond between positions 66 and 67 of A1-β-casein in comparison to the proline–isoleucine bond between positions 66 and 67 of A2-β-casein. BCM-7 is enzymatically cleaved between positions 60 and 66 [1,2].

Raw A1A1 and A2A2 cow’s milk contains comparably low BCM-7 concentrations of 2.0 to 3.0 ng/mL [4]. However, during digestion or processing, the BCM-7 content increases, particularly in A1A1 or A1A2 milk or milk products [1,2,3]. In vitro, an increase in BCM-7 was observed during gastrointestinal digestion experiments using casein and digestive enzymes such as pepsin or pancreatic elastase [4,5,6]. Asledottir et al. [6] showed in their in vitro study that BCM-7, treated with human gastrointestinal (GI) juices and porcine brush border membrane, was further degraded: 79% of the peptide was cleaved after 2 h, but after 24 h still 5% of the initial BCM-7 content could be determined [6]. Compared to raw milk, higher BCM-7 contents are found in milk products such as cheeses, due to the proteolytic enzymes of the starter culture bacteria added or after the addition of gastric enzymes [7,8,9,10].

BCM-7 is hypothesized to act as an exogenous µ-opioid receptor agonist [1,2,3,11]. However, in an epidemiological report by the European Food Safety Authority (EFSA), the authors stated that BCM-7 is only a low-potent ligand to this receptor [12]. As due to the broad distribution of the µ-opioid receptors, numerous organs and tissues could be affected, with gastrointestinal dysfunctions such as flatulence, abdominal pain and altered stool consistency/frequency being most prominent [1,5,13]. Studies linking A1 milk and milk product consumption to diseases such as arteriosclerosis/cardiovascular disease, sudden infant death syndrome, autism and type I diabetes mellitus have also been published. Based on the evaluation of the EFSA panel, the evidence is quite low, and “a cause–effect relationship between the oral intake of BCM-7 or related peptides and etiology or course of any suggested non-communicable diseases cannot be established”, independent of gastrointestinal alterations [12]. Several reviews have been published stating links between A1 milk and milk product consumption and certain diseases, evidently with regard to gastrointestinal alterations [1,2,3,4,13,14,15]. However, considering these reviews, the EFSA report and other publications from the databases, no results have been published that analyzed, for example, the in vivo degradation of β-casein and the formation of BCM-7 in the human GI tract, or the intestinal resorption and distribution of the peptide in humans (toxicodynamic). Only the above-described negative effects of BCM-7 within the GI tract have evidently been shown [1,2,3,4,13,14,15].

Food safety is a critical public health concern, and measures to reduce the potential health risks associated with A1-β-casein are of advantage. One approach would be the setup of regulatory limits for BCM-7 in milk and dairy products. However, this is currently not applicable due to missing toxicodynamic data linking specific BCM-7 levels to negative health outcomes. Currently the majority of dairy cows in Europe and in other countries (e.g., Canada, New Zealand) show the homozygous A1A1 and heterozygous A1A2-β-casein genotype. These animals produce milk exclusively with A1-β-caseins (A1A1) or varying proportions of A1- and A2-β-caseins (A1A2) [2]. Thus, a strategy to reduce A1-β-casein could involve selective breeding to increase the A2A2 genotype in dairy cow populations, resulting in milk containing only A2-β-caseins [2]. However, transitioning to A2A2 genotypes would take long and pose economic challenges for cattle farmers. However, studies indicate that adverse effects on the milk yield and milk composition are not likely, as already published yield and composition results are mainly similar between the homozygous A1A1- and A2A2-β-casein genotypes [16,17,18,19], partly with higher milk yields in the A2A2 genotype [16,17].

In addition to the BCM-7 issue, another important difference is the varying technological properties of A1 and A2 milk. For example, A2 milk treated with acid or rennet showed lower firmness and longer gelation/coagulation times [20,21,22,23,24]. These differences are partially accompanied by varying physicochemical or sensory properties of cheeses or yogurts made from A1 and A2 milk [20,23,25,26,27,28,29]. For example, Nguyen et al. [20] found in A1A1 and A2A2 yogurt no significant differences in the pH and textural values, whereas Daniloski et al. [28] described a softer texture of A2A2 yogurt compared to the A1A1 product. Vigolo et al. [27] and Daniloski et al. [29] found no significant differences in the protein, fat, moisture, pH and color results between A1A1 and A2A2 cheese, whereas Gai et al. [23] determined significantly higher protein, lightness and redness of A2A2 cheese and higher hardness of cheese produced with A2A2 milk and ripened for 90 and 180 days. Cattaneo et al. [26] found higher protein and fat contents in A2A2 grana cheese, ripened for 3 or 6 months. In sensory analyses, Mendes et al. [25] showed no significant effects of the β-casein genotype on the flavor, texture and overall acceptance of a Petit Suisse cheese, but the sensory panel described the A2A2 Minas Frescal cheese as softer and creamier and the A1A1 cheese as firmer and drier.

Cheese production mainly involves coagulation, dewatering and refining [30]. The coagulation step is initiated by rennet and/or acid treatment of the milk, concentrating casein and fat while other components, mainly water, are removed with the whey (syneresis) [30]. This process of gel or curd formation is influenced by factors such as pH, calcium concentration, protein content and temperature [30,31]. For the production of low-moisture cheeses like semi-hard or hard cheese, rennet is usually used, as acid treatment results in higher moisture contents [31]. The pH in rennet-treated cheeses is mainly influenced by the metabolism of starter culture bacteria and the formation of lactic acid [31]. Depending on the cheese type, the gel is subsequently cut into small particles, pressed, formed, stored in salt and coated, followed by a ripening period [30,31]. During the different stages of cheese-making, there is a concomitant expulsion of moisture (whey) and an increase in the firmness of the cheese, again depending on the cheese type [31].

According to the German regulations on cheese, semi-hard cheeses show a water content in the fat-free cheese mass of 54% to 63%. Depending on the specific type, they are ripened for a period of 1 to 6 months. Prominent examples are Appenzeller (origin: Switzerland), Edamer and Gouda (origin: The Netherlands). Some varieties are characterized by a complex microbiota on their surface, which is achieved by smear-ripening [32].

The microbiological properties of cheeses are principally influenced by the (deliberate) addition of starter and ripening cultures, composed of lactic acid bacteria and others, and the (undesirable) contamination of the milk, curd or cheese at any production stage [33]. The starter cultures are added to generate lactic acid and enable syneresis, whey expulsion and curd formation, thereby mainly influencing flavor development [34]. Lactic acid bacteria are described as Gram-positive, acid-tolerant, fermentative microorganisms. Well-known lactic acid bacteria often used as starter or ripening cultures in semi-hard cheese production are *Lactococcus lactis* subsp. *lactis* or *cremoris*, *Lactobacillus casei*, *Lactobacillus helveticus*, *Streptococcus thermophilus*, *Propionibacterium freudenreichii* and *Brevibacterium linens* [35]. In addition to these deliberately applied cultures, the microbial content of the cheeses depends on factors such as the (udder) health of the cows, the milking process, the heating procedure of the raw milk, if applied, as well as further processing procedures, partly shown above [33,36]. Next to beneficial microbiota, raw milk can contain spoilage or even pathogenic microorganisms, depending on the health condition of the animal as well as the milking process. Regarding product spoilage, heat-resistant proteases and lipases, which are produced by psychrotrophic bacteria, are of great importance [37]. Proteases preferentially attack casein instead of whey proteins, with β-casein and κ-casein being more susceptible than α-casein [38]. Lipases break down triglycerides and thus cause flavor defects [39]. Psychrotrophic microorganisms isolated from milk include Gram-negative (*Pseudomonas*, *Aeromonas*, *Serratia*, *Acinetobacter*, *Alcaligenes*, *Achromobacter*, *Enterobacter* and *Flavobacterium*) and Gram-positive (*Bacillus*, *Aneurinibacillus*, *Brevibacillus*, *Geobacillus*, *Clostridium*, *Corynebacterium*, *Microbacterium*, *Micrococcus*, *Streptococcus*, *Arthbacter*, *Carnobacterium* and *Staphylococcus*) bacteria. Of these, *Pseudomonas* is the most frequently isolated genus [39]. In addition, dairy products, and cheese in particular, are repeatedly associated with foodborne outbreaks. Milk-borne diseases account for approx. 4% of all foodborne diseases worldwide [36]. Here, the most important pathogens are *Listeria monocytogenes*, Staphylococcus aureus, Shiga-toxin-producing Escherichia coli and Campylobacter [36,40]. Although a more intense and rich flavor is attributed to cheeses made from raw compared to pasteurized milk [33], the majority of manufacturers choose pasteurization. Treatment of raw milk for 15 s at 72 °C or 30 min at 63 °C, according to the European hygiene regulation (EC) No 853/2004, significantly reduces the microbial load of the milk with the exception of spore-forming bacteria such as *Bacillus* spp. [33,34,36]. This in turn significantly reduces cases of illnesses and hospitalizations due to consumption [36].

Nevertheless, pathogens can be transmitted at any stage of production during cheese-making, ripening and storage. Some pathogens, such as *L*. *monocytogenes* or *Bacillus cereus*, are able to form biofilms and to persist on food contact surfaces for a long time. Further, equipment as well as the food-handling staff can also contribute to cross-contamination, especially when unsafe handling and improper storage conditions take place [36,41]. In addition to the initial heating of the milk, which already significantly reduces germ counts, various preservation strategies such as the use of food additives, high pressure, edible coatings, vacuum, modified atmosphere or intelligent packaging are applied to improve the quality and shelf life of semi-hard cheeses [32,42].

Despite extensive research on BCM-7 and technological properties, the impact of the β-casein genotype on the physicochemical and microbiological properties of cheese throughout production, ripening and storage remains unexplored. This study aims to address this gap by analyzing cheeses made from pure A1A1 and A2A2 milk with regard to their physicochemical properties during production, ripening and modified atmosphere storage and their microbiological properties during the storage period. The investigations were completed by analyses of the BCM-7 concentrations throughout the production and ripening of the cheeses.

## 2. Materials and Methods

### 2.1. Material

In six independent experiments, at least 5 to 6 L of raw milk was collected from the Lehr- und Forschungsgut Ruthe of the University of Veterinary Medicine Hannover, Foundation, and transported at 7 °C to the Institute of Food Quality and Food Safety. The raw milk was collected from up to four cows of the German Holstein breed with the homozygote β-casein genotypes A1A1 and A2A2, molecular biologically verified by the Lehr- und Forschungsgut. The state of lactation of the animals was 2 ± 1 months after birth.

### 2.2. Cheese Production, Packaging and Sample Collections

#### 2.2.1. Cheese Production, pH Analysis

At least 24 h after collection of the raw milk, the milk was pasteurized two times for 30 min at 65 °C in a water bath with a shaker function (GFL, Lauda-Königshofen, Germany) and subsequently cooled down to room temperature.

The pasteurized milk was used for the production of cheeses using two identical cheese sets (MilkySky GmbH, Lauben, Germany). A total of 2.5 L of the pasteurized milk per cheese set was warmed to 32 °C, and 0.5 mL of calcium chloride (0.6 mM; MilkySky GmbH, Lauben, Germany) was added. Then the starter culture mixture was added (Selection Danica, Chr. Hansen GmbH, Nienburg, Germany) considering the specifications of the producer (0.25 units/2.5 L). The starter culture mixture consists of the bacteria species *Lactococcus lactis* subsp. *cremoris*, *Lactococcus lactis* subsp. *lactis*, *Lactococcus lactis* subsp. *lactis* biovar. Diacetylactis, *Leuconostoc mesenteroides* subsp. *mesenteroides*, *Leuconostoc mesenteroides* subsp. *cremoris* and *Leuconostoc pseudomesenteroides*.

After an incubation period of 45 min at 32 °C, microbial rennet (Microlant Classic 200, Chr. Hansen, Nienburg, Germany) was added considering the specifications of the producer. The rennet consists of an aspartic protease (mucorpepsin), produced by *Rhizomucor miehei*, and was added at a concentration of 60 international milk-clotting units per L of milk.

After a further incubation at 32 °C for 30 min, the curd was carefully cut into cubes with a size of 0.5 × 0.5 × 0.5 cm^3^. The temperature was kept at 32 °C and the curd was carefully stirred every 10 min.

Forty-five minutes after the first cutting, the curd was washed by removing 600 mL of whey from every cheese set and replacing it with the same amount of hand-warm water. This mixture was again incubated at 32 °C for 45 min.

Ninety minutes after the first cutting, the curd–whey mixture was heated to 38 °C and 1.2 kg was transferred to four cylindrical cheese forms (MilkySky GmbH, Lauben, Germany). Within the forms, the mixture was pressed with precisely fitting cylindrical weights (1.5 kg) for 16 h. After 30 min, 45 min, 60 min and 16 h, the cheeses were turned within the forms.

On the next day, the cheese loaves were transferred to a salt bath, which consisted of 25% whey, 0.375 g/L sea salt and 0.04% lactic acid (MilkySky GmbH, Lauben, Germany). After incubation for 16 h at 12 °C, the loaves were removed from the salt bath and dried for 3 h at room temperature.

The loaves were coated with a non-edible plastic coating (MilkySky GmbH, Lauben, Germany). The coated cheeses were then ripened for 6 weeks at 13 °C.

In the raw and pasteurized milk, after the addition of the starter culture mixture, after pressing, after the salt bath and after ripening for 2, 4 and 6 weeks, the pH values were determined. The color values were additionally determined after pressing, after the salt bath and after ripening for 2, 4 and 6 weeks.

#### 2.2.2. Packaging and Storage

After 6 weeks of ripening, a part of the cheeses was cut into slices with a thickness of 3 mm and packed in modified atmosphere packages (MAP, 70% N_2_, 30% CO_2_) using the packaging machine T100 (Multivac Sepp Haggenmüller, GmbH & Co. KG, Wolfertschwenden, Germany). The MAP was stored up to day 84 at 4 °C. The MAP was opened on days 0 (day of packaging), 21, 42, 63 and 84, and immediately 10 g was removed for the microbiological analysis of the total viable counts (TVCs). Then the color values were determined on the surface of the cheese slices.

#### 2.2.3. Sample Collection

A sample for the analysis of the activity of the alkaline phosphatase was collected after the second pasteurization step to verify the successful pasteurization process.

For the analysis of the BCM-7 concentrations, each 100 mL of the raw and pasteurized milk and each 175 g of the cheese after pressing, after the salt bath and after 2 and 6 weeks of ripening were collected, frozen and stored at −20 °C for further analyses. The cheese samples were homogenized for 1 min at 10,000 rpm (Grindomix GM 200, Retsch GmbH, Haan, Germany) before the freezing step.

A part of the homogenate was used for analysis of the water activities (a_w_).

After 6 weeks of ripening, a part of the homogenized sample was used for the analysis of fat, protein, NaCl and dry matter.

At the end of the ripening period, at week 6, in three of the six independent experiments, samples of the cheeses were collected for determination of the hardness values.

### 2.3. Analytical Methods

#### 2.3.1. Physicochemical Parameters

The activity of the alkaline phosphatase was determined with the Lactognost^®^ test (Heyl, Berlin, Germany), as described by the manufacturer. The alkaline phosphatase is inactivated during pasteurization. In brief, in the Lactognost^®^ test, the enzymatically active alkaline phosphatase degrades phenyl phosphate disodium salt, thereby releasing phenol. Phenol reacts with 2,6-dibromoquinone-4-chloroimide at a pH of 9.0 to a blue reaction product. If the alkaline phosphatase is inactivated, the solution is reddish brown.

The pH values of the cheeses were determined with a pH meter (Portamess^®^, Knick GmbH, Berlin, Germany) equipped with a temperature sensor and a glass electrode (InLab 427, Mettler-Toledo, Urdorf, Switzerland). Before measurement, the apparatus was calibrated with standard solutions (pH 4.0, pH 7.0, Carl Roth GmbH & Co. KG, Karlsruhe, Germany). The pH value was determined in duplicates. The duplicate and the mean value were used for statistical analysis.

The water activity (a_w_) of the homogenized cheese samples was determined once with an a_w_-cryometer (AWK-40, NAGY Messsysteme GmbH, Gäufelden, Germany) after checking the correct functionality with a 5% KCl solution.

With a colorimeter (Konica-Minolta GmbH, Langenhagen, Germany, 8 mm measuring field, standard observer, D65 illuminant), the lightness (L*), redness (a*) and yellowness (b*) of the cheeses were determined. The apparatus was calibrated with a white plate (Konica-Minolta GmbH, Langenhagen, Germany). The color was measured three times, and for statistical analysis, the mean value was used.

The hardness (in Newtons, N) of the cheeses was analyzed using a texture analyzer, Ta.XT.plus (Stable Micro Systems, Survey, UK). The apparatus consists of a 50 kg power cell and a round aluminum stamp (50 mm), which moved down during every experiment with a speed of 3 mm/s until 40% of the sample height was reached and finally up again with a speed of 3 mm/s. For the measurements, which were performed in triplicates, the cheeses were cut to a thickness of 25 mm and a diameter of 22 mm. The mean values of the triple analysis were used for statistical analysis.

Using a near-infrared apparatus (NIR, model TANGO, Bruker corporation, Leipzig, Germany), the contents of protein, fat, NaCl and the dry matter of the homogenized cheeses, collected on week 6 of ripening, were determined.

#### 2.3.2. Microbiological Parameters

For analysis of the total viable number (TVC) and the number of *Lactobacillus* spp. during storage in MAP, 10 g of the cheese slices were weighed, filled up with NaCl–Pepton (0.85% NaCl, 0.1% Pepton; VWR, Darmstadt, Germany) to the ninefold (90 g) and homogenized at 230 rpm for 2 min with a stomacher (Stomacher 400 Circulator, Seward limited, Worthing, UK). Subsequently, serial dilutions up to 10^6^ were prepared. For analysis of the TVC, each 1 mL of the appropriate dilutions was pipetted into a sterile petri dish, and 15 mL of plate count agar (Oxoid GmbH, Wesel, Germany) was added. After the agar had solidified, the plates were incubated at 30 °C for 72 h [43]. For analysis of *Lactobacillus* spp., 0.1 mL of the appropriate dilutions were pipetted on MRS agar (Oxoid GmbH, Wesel, Germany), distributed with a sterile spatula and incubated for 72 h at 25 °C. After incubation, visible colonies were counted, and the number of colony-forming units (cfus) per g of cheese was calculated. The detection limits for the TVC were 1.0 log_10_ cfu/ g cheese, and for the lactobacilli, 2.0 log_10_ cfu/ g cheese. Half of these values (0.7 or 1.7 log_10_ cfu/ g cheese) were used for further calculations if no colonies were found on the agar plates.

#### 2.3.3. BCM-7 Determination

For determination of the BCM-7 concentration in cheese, samples were homogenized with water, centrifuged, acidified and filtered prior to LC-MS^3^ analysis. In detail, 5 g of each cheese sample were homogenized in 10 mL of purified water using a blender (MICCRA GmbH, Buggingen, Germany). The suspension was incubated in a water bath at 40 °C for 1 h. Afterwards, samples were centrifuged at 10,000× *g* and 10 °C for 30 min. Supernatant was collected and acidified to pH 4.6 using 2 M hydrochloric acid (Carl Roth GmbH & Co. KG, Karlsruhe, Germany). Again, samples were centrifuged at 5000× *g* and 4 °C for 20 min. At least 1 mL of the supernatant was filtered using 2.0 µm Millex^®^-AP borosilicate glassfiber AP20 (Merck KGaA, Darmstadt, Germany) and subsequently 0.2 µm Rotilabo^®^ Mini-Tip syringe (Carl Roth GmbH & Co. KG, Karlsruhe, Germany) filters. After this step, samples were frozen and stored until transport on dry ice to the Institute of Food Chemistry, Münster. Directly prior to LC-MS^3^ analysis, 10 µL of internal standard solution (2.37 mg/mL of ([^13^C_5_;^15^N]Pro^2,4,6^)-β-casomorphin in purified water) was added to 90 µL of each sample. A total of 20 µL was injected onto a Nucleoshell C18 Bluebird column (2.0 × 100 mm, 2.7 µm, Macherey-Nagel, Düren, Germany) equipped with a 2.0 × 4.0 mm guard column of the same material. For separation, a 1260 Infinity UHPLC system (Agilent, Waldbronn, Germany) was used. A binary gradient consisting of acetonitrile (A) and water (B), both supplemented with 0.1% formic acid, was applied at a flow rate of 300 µL/min. Starting from 5% A for the first 1.5 min, the gradient went to 100% A after 12.5 min. These conditions were maintained for 2.5 min, followed by 4.5 min of re-equilibration at 5% A. For detection, the QTRAP 6500 mass spectrometer (Sciex, Darmstadt, Germany) equipped with a positive ESI Turbo V ion source at 5500 V and a 500 °C heater temperature was used. Curtain gas was set at 50 psi, nebulizer gas was set at 45 psi, heating gas was set at 55 psi, declustering potential was set at 80 V and entrance potential was set at 10 V. The mass spectrometer was operated in MS^3^ mode at a scan rate of 10,000 Da/s and a fill time of the linear ion trap of 50 ms followed by excitation for 20 ms. For BCM-7, *m*/*z* 790.4 was the first precursor ion, and after fragmentation in the collision cell with a potential of 43 V, *m*/*z* 383.2, the precursor of the MS^3^ experiment was obtained. Product ions of *m*/*z* 229.1 and *m*/*z* 286.1 were used as quantifier and qualifier, respectively. For isotope-labeled BCM-7, *m*/*z* 808.5 was the first precursor, and *m*/*z* 395.3 was the second, produced by a collision energy voltage of 40 V. Product ions of *m*/*z* 235.1 and *m*/*z* 292.1 were used as quantifier and qualifier, respectively. BCM-7 concentration was determined by internal calibration using calibration series of BCM-7 in water.

### 2.4. Statistical Analysis

For statistical analysis, SAS Enterprise Guide 7.1 (SAS Institute Inc., Cary, NC, USA) was used. The data were analyzed with the mixed model, considering the following models:

$$Y_{ij}=µ+C_{i}+R_{i}+\varepsilon_{ij}$$

Y_ij_ = observation value; µ = overall mean, C_i_ = fixed effect of the β-casein genotype (A1A1, A2A2); R_j_ = random effect of repeat; ε_ij_ = random error.

If the F test was significant (*p* ≤ 0.05), the individual significant differences were calculated with the Tukey multiple comparison test. Differences between the groups were significant if the *p*-value in the Tukey test was lower than 0.05.

## 3. Results

### 3.1. Physicochemical Results

#### 3.1.1. Alkaline Phosphatase, pH and a_w_ Values

Alkaline phosphatase activity of all milk samples was negative after pasteurization, indicating a successful heating process.

**Figure 1:**
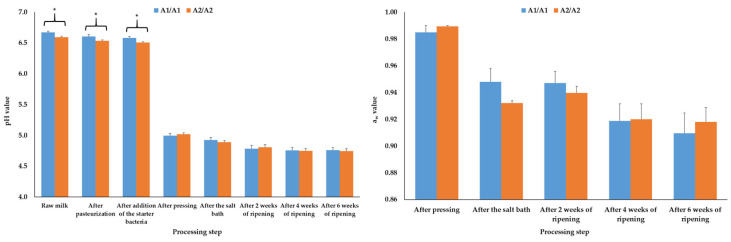
Mean and standard error values of the pH (**left**) and water activity (a_w_, (**right**)) results of the cheeses depending on the β-casein genotype of the raw milk and the step during production of the cheese (N = 6); * mark significant differences between the β-casein genotypes (*p* ≤ 0.05).

The pH values during the production of the A1A1 cheeses were significantly (*p* ≤ 0.05) higher in the raw milk, after pasteurization and after the addition of the starter culture compared to the A2A2 products, whereas during further processing the pH values were comparable (*p* > 0.05) between the cheeses of the two groups (Figure 1, left).

Considering the water activity (a_w_) values, which were determined at first after pressing of the cheeses, no significant effect (*p* > 0.05) of the β-casein genotype could be found (Figure 1, right).

#### 3.1.2. Color Values

**Figure 2:**
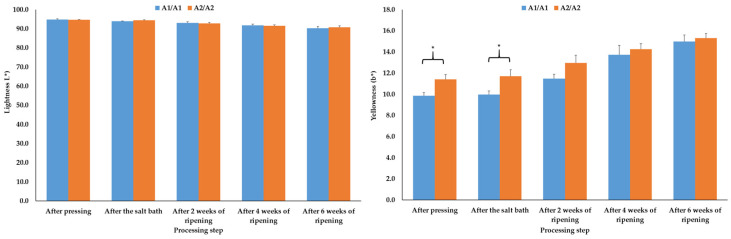
Mean and standard error values of the lightness (L*, (**left**)) and yellowness (b*, (**right**)) results of the cheeses depending on the β-casein genotype of the raw milk and the step during production of the cheese (N = 6); * mark significant differences between the β-casein genotypes (*p* ≤ 0.05).

The lightness and yellowness values, which were determined at first after pressing of the cheeses, were comparable (*p* > 0.05) between the cheeses of the two β-casein genotype with two exceptions. After pressing and after the salt bath, the A1A1 cheeses showed significantly (*p* ≤ 0.05) lower yellowness values compared to the cheeses produced from A2A2 raw milk (Figure 2). The redness results were also comparable (*p* > 0.05) at all processing steps analyzed. The a* values were, depending on the processing step, between −1.13 and −1.52 in the A1A1 cheeses and between −1.16 and −1.36 in the A2A2 products. 

**Figure 3:**
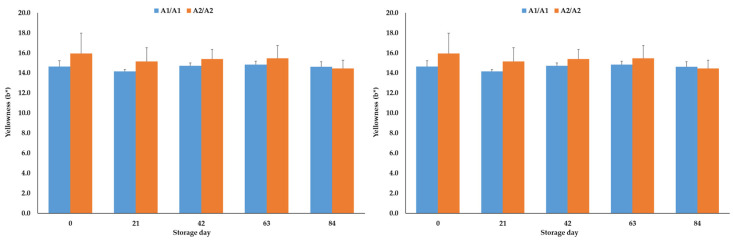
Mean and standard error values of the lightness (L*, (**left**)) and yellowness (b*, (**right**)) results of the cheeses, ripened for 6 weeks and then packaged in modified atmosphere packages (30% CO_2_, 70% N_2_), depending on the β-casein genotype of the raw milk and the storage day of the cheese (N = 3).

During storage of the cheese slices up to day 84, the lightness and yellowness values were not significantly (*p* > 0.05) influenced by the β-casein genotype of the raw milk used for the production of the cheeses (Figure 3). The redness results of the A1A1 and A2A2 cheese slices were also comparable (*p* > 0.05) during the modified atmosphere storage up to day 84. The values were, depending on the storage day, between −1.5 and −1.9 in the A1A1 cheeses and between −1.5 and −2.2 in the A2A2 products. 

#### 3.1.3. Texture, Fat, Protein, NaCl and Dry Matter Values

The hardness, fat, protein, NaCl and dry matter values of the cheeses, which were determined after 6 weeks of ripening, were comparable (*p* > 0.05) between the cheeses of the two β-casein genotypes. However, the hardness values were tendentially (*p* = 0.106) higher in the A2A cheeses compared to the A1A1 products (Table 1).

### 3.2. Microbiological Results

**Figure 4:**
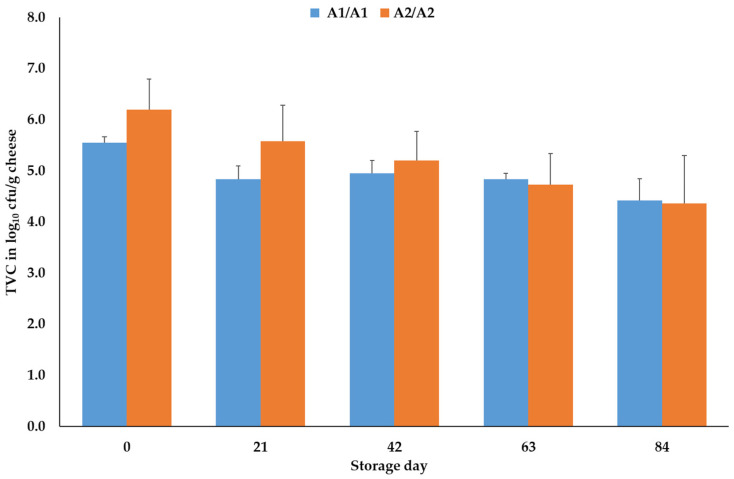
Mean and standard error values of the total viable count (TVC) results (in log_10_ colony-forming units (cfus)) of the cheeses, ripened for 6 weeks and then packaged in modified atmosphere packages (30% CO_2_, 70% N_2_), depending on the β-casein genotype of the raw milk and the storage day of the cheese (N = 3).

During storage of the cheese slices up to day 84, the TVC values and the number of lactobacilli were not significantly (*p* > 0.05) influenced by the β-casein genotype of the raw milk used for the production of the cheeses (Figure 4, only TVC).

### 3.3. BCM-7 Results

**Figure 5:**
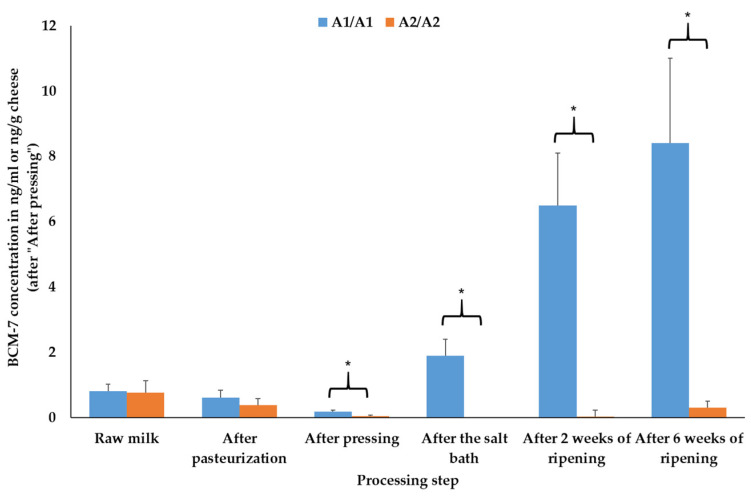
Mean and standard error values of the β-casomorphin-7 (BCM-7) results of the cheeses depending on the β-casein genotype of the raw milk and the processing step of the cheese in modified atmosphere packages (30% CO_2_, 70% N_2_, N = 3); * mark significant differences between the β-casein genotypes (*p* ≤ 0.05).

The concentrations of BCM-7 were significantly higher (*p* ≤ 0.05) after pressing, after the salt bath and after ripening for two and six weeks in the cheeses produced with A1A1 raw milk (Figure 5).

## 4. Discussion

This study investigated the formation and degradation of BCM-7 in cheeses made from A1A1 and A2A2 raw milk. Additionally, physicochemical and microbiological parameters of these cheeses during production, ripening and storage were examined in up to six independent experimental repetitions. Homozygous genotypes (A1A1 and A2A2) were selected to ensure clear and interpretable results. In contrast, heterozygous A1A2 genotypes produce milk containing a mixture of A1- and A2-β-casein molecules [1,2], likely resulting in intermediate and less conclusive outcomes. This assumption is supported by studies that analyzed the BCM-7 concentrations after in vitro gastrointestinal digestion experiments [4] and the physicochemical properties of cheese and yogurt produced with milk of the three genotypes [23,27,28,29].

The pH values determined in this study align with previous findings [22,28,30,31]. In agreement with Daniloski et al. [28], lower pH values were observed in the A2A2 milk at the beginning of the cheese production. Juan et al. [22] detected comparable pH values in A2A2 cow’s milk in comparison to a mixture of A1A1, A2A2 and A1A2 milk. Differences in pH may be attributed to the amino acids at position 67 in the A1- and A2-β-casein molecules, which influence the secondary protein structure and hydrophobicity of the proteins [1,2,21]. The pH differences observed in the present study might explain the varying coagulation properties of A1 and A2 milk, as A2 milk shows lower firmness and longer gelation/coagulation times if treated with acid or rennet [20,21,22,23]. The isoelectric pH influences casein aggregation after acid and rennet treatment of the milk [30,31]. However, a study by Hallen et al. [44] showed comparable pH values in milk with good and poor coagulating properties, and Macciotta et al. [45] found no correlation between the pH values and the curd firming time and curd firmness. The determined comparable pH values after the addition of the starter culture are principally consistent with investigations by Gai et al. [23], Vigolo et al. [27] and Daniloski et al. [29], who analyzed the cheese after production/ripening. The latter result is comprehensible, as the pH development after this production step is mainly influenced by the metabolism of the bacteria added and their lactic acid production [33,34,35].

Water activity (a_w_) is an index for the presence of free water in cheese, primarily influenced by the ripening time as well as water, water-soluble protein and salt (NaCl) content of the cheese [46,47]. Fresh curd has a high a_w_ value of 0.98 to 0.99 due to water entrapped in the casein–fat network [47]. During further processing and ripening/drying of the cheese, the a_w_ values reduce as free water is expelled from this network [46,47]. This decrease was also observed in the present study, but no significant differences in a_w_ were found between A1A1 and A2A2 products. As water activity values of cheeses made from A1A1 and A2A2 milk have not been published, a discussion is challenging. However, the results of the present study are plausible since the water, protein and NaCl results were similar after six weeks of ripening.

The mainly comparable color values of the A1A1 and A2A2 cheeses during ripening and vacuum storage for 84 days agree with the study of Daniloski et al. [29], who also found similar color values of A1A1 and A2A2 cheeses. However, Gai et al. [23] determined higher L* values in A2A2 cheeses compared to the A1A1 products. Cheese color is influenced by several factors, such as the feeding or the lactation stage, which alter milk and cheese composition [48,49]. In his study, milk was sourced from cows with identical feeding regimes and comparable states of lactation. Additionally, the fat, protein and dry matter results after six weeks of ripening were comparable, potentially explaining the comparable color results of the cheeses in the present study. This assumption of a composition/color relation is supported on the one hand by the results of Daniloski et al. [29], who found, in addition to similar color values, also comparable fat, protein and moisture values of A1A1 and A2A2 cheeses, and on the other hand by the study of Gai et al. [23], who found, in addition to higher L* values, higher protein contents in the A2A2 cheeses compared to the A1A1 products.

Interestingly, significantly higher yellowness of the A2A2 products could be determined after pressing and after the salt bath. In the study by Wang et al. [50], the b* values of fermented A1 and A2 milk also differed significantly, with higher b* values of the A2 milk after fermentation. However, the data in the present study should not be overestimated, as the yellow values became similar as the cheeses ripened.

The comparable hardness results agree with the publications of Gai et al. [23] and Daniloski et al. [29], considering their results from 30 days of ripening time. However, longer ripening periods (180 days) have been associated with higher hardness values in A2A2 cheeses compared to A1A1 products [23,29]. Daniloski et al. [29] showed that after 180 days of ripening, the structure of A2A2 cheese was microscopically less porous (more compact) compared to the A1A1 cheeses, which explains the higher hardness results of the A2A2 products. A relation of the differing structure to the varying rennet coagulation properties of the A1 and A2 milk might be the reason [20,21,22,23].

The total viable counts (TVCs) and *Lactobacillus* spp. results primarily reflect the activity of starter cultures, which drive the biochemical reactions necessary for transforming milk and curd into ripened cheese. As the lactobacilli influence the color of A1 and A2 milk during fermentation [50], we also wanted to evaluate if the microbiological properties of cheeses made of A1 and A2 milk differ during the storage of the 6-week-ripened products. To our knowledge, no previous studies have investigated the impact of β-casein genotype on cheese microbiology, making direct comparisons difficult. Anyway, the result of the present study is that the different β-casein variants do not influence the growth of bacteria is negotiable, as bacterial growth in cheese is mainly influenced by pH, a_w_, water and nutrients [51], factors that were comparable between the A1A1 and A2A2 cheeses. Further investigations are in progress to evaluate the microbiological parameters during the production and ripening of the A1 and A2 cheeses.

Finally, the higher BCM-7 concentrations in the cheeses made from A1A1 milk align with previous publications and reviews [1,2,3,4,5,7,8,9]. A1- and A2-β-casein proteins differ in one amino acid at position 67 of the primary structure. At this position, A1-β-casein contains histidine, while A2-β-casein contains proline [2]. As the histidine–isoleucine bond in A1-β-casein is more prone to enzymatic cleavage (position 66–67), higher BCM-7 release is observed compared to A2-β-casein with the more stable proline–isoleucine bond [1,2].

## 5. Conclusions

The use of A1A1 and A2A2 milk for the production of a semi-hard cheese resulted in no differences in the majority of the physicochemical and microbiological parameters analyzed during the different production and ripening steps over a period of up to six weeks or during the storage of cheese slices in modified atmosphere packages for 84 days. These results indicate that neither the A1A1 nor the A2A2 cheese can be associated with distinct or superior quality. However, certain results, such as the varying pH values of the raw, pasteurized and starter culture-treated milk or the differing yellowness results at the initial steps of cheese production, should be taken into account, particularly if cheese is not ripened (fresh cheese) or other products like yogurt are produced from A1A1 or A2A2 milk. While the increasing BCM-7 concentrations in the A1A1 cheeses are comprehensible, health-related problems of A1A1 cheese are difficult to assess, as limits and toxicodynamic results of BCM-7 are not applicable. Nevertheless, significant health concerns appear unlikely, as overall concentrations and the concentration differences between A1A1 and A2A2 cheeses are quite low.

## Figures and Tables

**Table 1:** Mean and standard deviation values of different physicochemical parameters depending on the β-casein genotype of the raw milk after ripening of the cheese for six weeks (N = 6, hardness (N = 3)).

Parameters	A1A1	A2A2
Hardness in N	8.72 ± 3.4	18.8 ± 5.4
NaCl (%)	3.75 ± 0.79	3.17 ± 1.02
Fat (%)	27.3 ± 5.9	27.8 ± 7.3
Protein (%)	24.9 ± 2.8	23.8 ± 2.4
Dry matter (%)	63.8 ± 9.3	62.9 ± 7.4

## Data Availability

The original contributions presented in this study are included in the article. Further inquiries can be directed to the corresponding author.
